# Clinical implication of voltage-dependent anion channel 1 in uterine cervical cancer and its action on cervical cancer cells

**DOI:** 10.18632/oncotarget.6704

**Published:** 2015-12-21

**Authors:** Chih-Hsien Wu, Yu-Wen Lin, Tzu-Fan Wu, Jiunn-Liang Ko, Po-Hui Wang

**Affiliations:** ^1^ Institute of Medicine, Chung Shan Medical University, Taichung, Taiwan; ^2^ Department of Obstetrics and Gynecology, Chung Shan Medical University Hospital, Taichung, Taiwan; ^3^ School of Medicine, Chung Shan Medical University, Taichung, Taiwan

**Keywords:** voltage-dependent anion channel 1, gene silencing, cell growth, mitochondrial membrane potential, uterine cervical cancer

## Abstract

Two-dimensional gel electrophoresis and liquid chromatography-tandem mass spectrometry were performed to investigate the influence of human nonmetastatic clone 23 type 1 (nm23-H1), a metastasis-associated gene on proteomic alterations in cancer cells of the uterine cervix. It was validated by RT-PCR and Western blot analysis. The expression of voltage-dependent anion channel 1 (VDAC1) was increased in nm23-H1 gene silenced SiHa or CaSki cervical cancer cells. The clinical implication was shown that cervical cancer tissues with positive VDAC1 immunoreactivity exhibited deep stromal invasion (>10 mm in depth) and large tumor size (> 4 cm in diameter). Cervical cancer patients with positive VDAC1 immunoreactivity displayed higher recurrence and poorer overall survival than those with negative VDAC1. Silencing of VDAC1 reduced cell proliferation and migratory ability. Mitochondrial membrane potential was decreased and reactive oxygen species generation was increased in the VDAC1 gene-silenced cervical cancer cells. Cell cycle progression and autophagy were not changed in VDAC1 silencing cells. The cytotoxicity of cisplatin was significantly enhanced by knockdown of cellular VDAC1 and the compounds that interfere with hexokinase binding to VDAC. Therapeutic strategies may be offered using VDAC1 as a target to reduce cell growth and migration, enhance the synergistic therapeutic efficacy of cisplatin and reduce cisplatin dose-limiting toxicity.

## INTRODUCTION

Our previous experiment, using genechips, revealed that human nonmetastatic clone 23 type 1 (nm23-H1) has decreased the expression of voltage-dependent anion channel 1 (VDAC1) in cancer cells of the uterine cervix. We previously demonstrated that knockdown of the nm23-H1 gene increased the proliferation of SiHa cancer cells [1]. Therefore, VDAC1 was hypothesized to be associated with the development and progression of cervical cancer.

VDAC1 is mainly located at the outer mitochondrial membrane (OMM) and is the most abundant protein in OMM. It forms a channel for the entry and exit of metabolites across the OMM [2–4]. Because VDAC1 can bind with pro-apoptotic [5–10] or anti-apoptotic proteins [11–14], it may stimulate or inhibit the apoptosis, growth and survival of cervical cancer cells respectively. Mitochondrial membrane permeability may be controlled by the mitochondrial permeability pore (PTP) [15].

The PTP is composed of or activated by VDAC1, adenine nucleotide translocase and cyclophilin in cancer cells [16]. The prolonged opening of the polyprotein complex has been shown to be accompanied by the dissipation of mitochondrial membrane potential (MMP) in many physiopathological models and may lead to cell death [15].

In human, three isoforms of VDAC1 have been identified (VDAC1-3) with different functions [17, 18]. Among these isoforms, VDAC1 has been shown to be the most abundant in HeLa cervical cancer cells [19].

The antitumor activity of DNA cross-linking agents including the most well known, cisplatin, is known to be attributable to their ability to prevent cell replication by forming intra- and inter-strand cross-links on nuclear DNA [20, 21]. In addition, methyl jasmonate, the plant stress hormone of the jasmonate family and clotrimazole have been shown to bind to hexokinase, which protects against cell death via interactions with VDAC1, and to detach it from mitochondria thus interfering with hexokinase binding to VDAC leading to an anti-tumor effect in several cancer cell types [22–24]. Therefore, these compounds probably enhance the therapeutic efficacy of conventional chemotherapeutic drugs and reduce dose-limiting toxicity.

In this study, we first established an association of the VDAC1with the prognosis in cervical cancer patients. We then investigated the role of VDAC1 in cell growth and the metastatic potential of uterine cervical cancer cells.

We examined the action by which VDAC1 exerted its effect on the cancer cells and investigated whether the presence of VDAC1 had correlations with MMP, cell cycle, autophagy protein expressions and reactive oxygen species (ROS). Finally, we investigated the influence of the reagents that detached hexokinase-VDAC binding and VDAC1 gene silencing on the cytotoxicity of cisplatin.

## RESULTS

### The effect of nm23-H1 on the expression of VDAC1 in uterine cervical cancer cells

**Figure 1:**
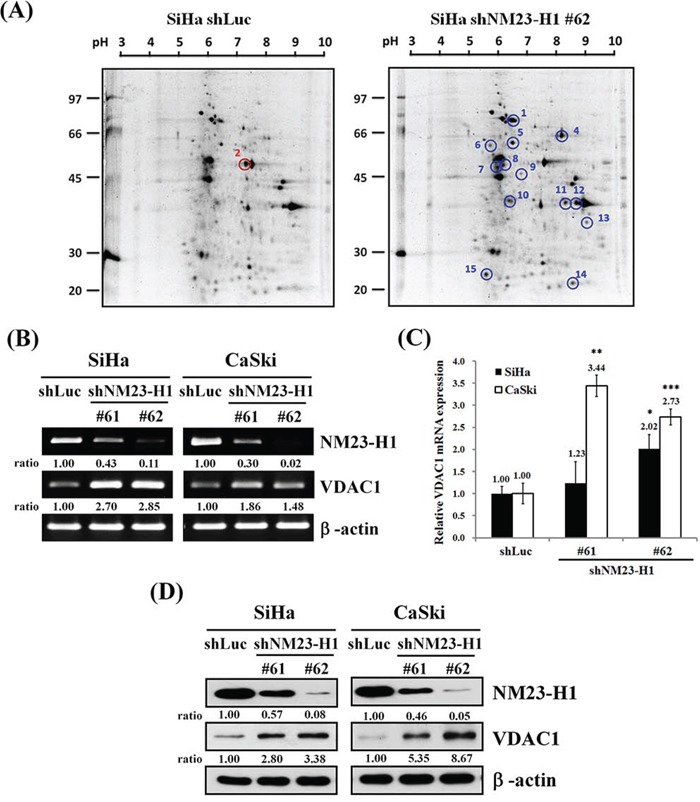
The influence of nn23-H1 on VDAC1 **A.** Two-dimensional gel analysis of the nm23-H1-correlated differentially expressed proteins. Equal amounts (150 μg) of cell lysates were separated on 11-cm (pH 3-10) linear gradient IPG strips using 10% SDS PAGE. A molecular marker was loaded on the left-hand side. It revealed elevated (blue circle) and reduced (red circle) expressions of some proteins after SiHa cancer cells of the uterine cervix were infected with lentiviruses carrying shnm23-H1 #62 compared to those with control vector (shLuc). The point 13 denotes VDAC1. SiHa and CaSki cells were infected with lentiviruses carrying shnm23-H1 #61, #62 or shLuc. The RNA levels were determined by RT-PCR **B.** and real-time PCR **C.** β-actin was used as the internal control. The relative ratios of nm23-H1/β-actin and VDAC1/β-actin are shown for RT-PCR. All values have been normalized to the level of β-actin and are the average of three independent readings for real-time PCR. **D.** Cells (5×10^5^ cells/6-cm dish) were seeded for 48 hours, and Western blotting was used to detect the protein levels of nm23-H1 and VDAC1 in SiHa or CaSki shnm23-H1 #61, #62 and shLuc cancer cells. The relative ratios of nm23-H1/β-actin and VDAC1/β-actin are shown. NM23-H1, human nonmetastatic clone 23 type 1; VDAC1, voltage-dependent anion channel 1. *p<0.05, **p<0.01, ***p<0.001.

After the nm23-H1 gene had been silenced by lentiviruses carrying shnm23-H1 #62, we identified and quantified some nm23-H1 associated proteins with high scores using 2D electrophoresis (Table 1). Among them, the VDAC1 protein level was found to be significantly increased (point 13 in Figure 1A). This was verified by an increased level of mRNA by both RT-PCR (Figure 1B) and quantitative real-time PCR (Figure 1C) after silencing of the nm23-H1 gene in SiHa and CaSki cancer cells. Western blotting also demonstrated an elevated expression of the VDAC1 protein in the nm23-H1 silenced cells (Figure 1D).

**Table 1:** Identification and quantification of NM23-H1 (human nonmetastatic clone 23 type 1)-associated proteins

Spot ID	Protein ID	Gene name	Score	no. of peptide	Accession No	PI	MW(KDa)
2	FLJ46536	RUN and FYVE domain containing 4 (RUFY4), transcript variant 1	14.07	2	38348282	7.06	44.8
4-1	pyruvate kinase isozymes M1/M2 isoform a	pyruvate kinase, muscle (PKM2), transcript variant 1	60.17	6	33286418	7.73	57.9
4-2	pyruvate kinase isozymes R/L isoform 1	pyruvate kinase, liver and RBC (PKLR)	20.15	2	10835121	7.61	61.8
5	protein disulfide-isomerase A3 precursor	protein disulfide isomerase family A, member 3 (PDIA3)	60.18	6	21361657	5.95	56.7
11	aldo-keto reductase family 1 member C3	aldo-keto reductase family 1, member C3 (3-alpha hydroxysteroid dehydrogenase, type II) (AKR1C3)	30.13	3	24497853	7.89	36.8
12	PHD finger protein 7 isoform 1	PHD finger protein 7 (PHF7)	14.05	2	21361543	8.03	43.7
13	voltage-dependent anion-selective channel protein 1	Homo sapiens voltage-dependent anion channel 1 (VDAC1)	20.17	2	4507879	8.86	30.8

ID: identification; PI: isoelectric point

### The clinical implication of VDAC1 in uterine cervical cancer

**Figure 2:**
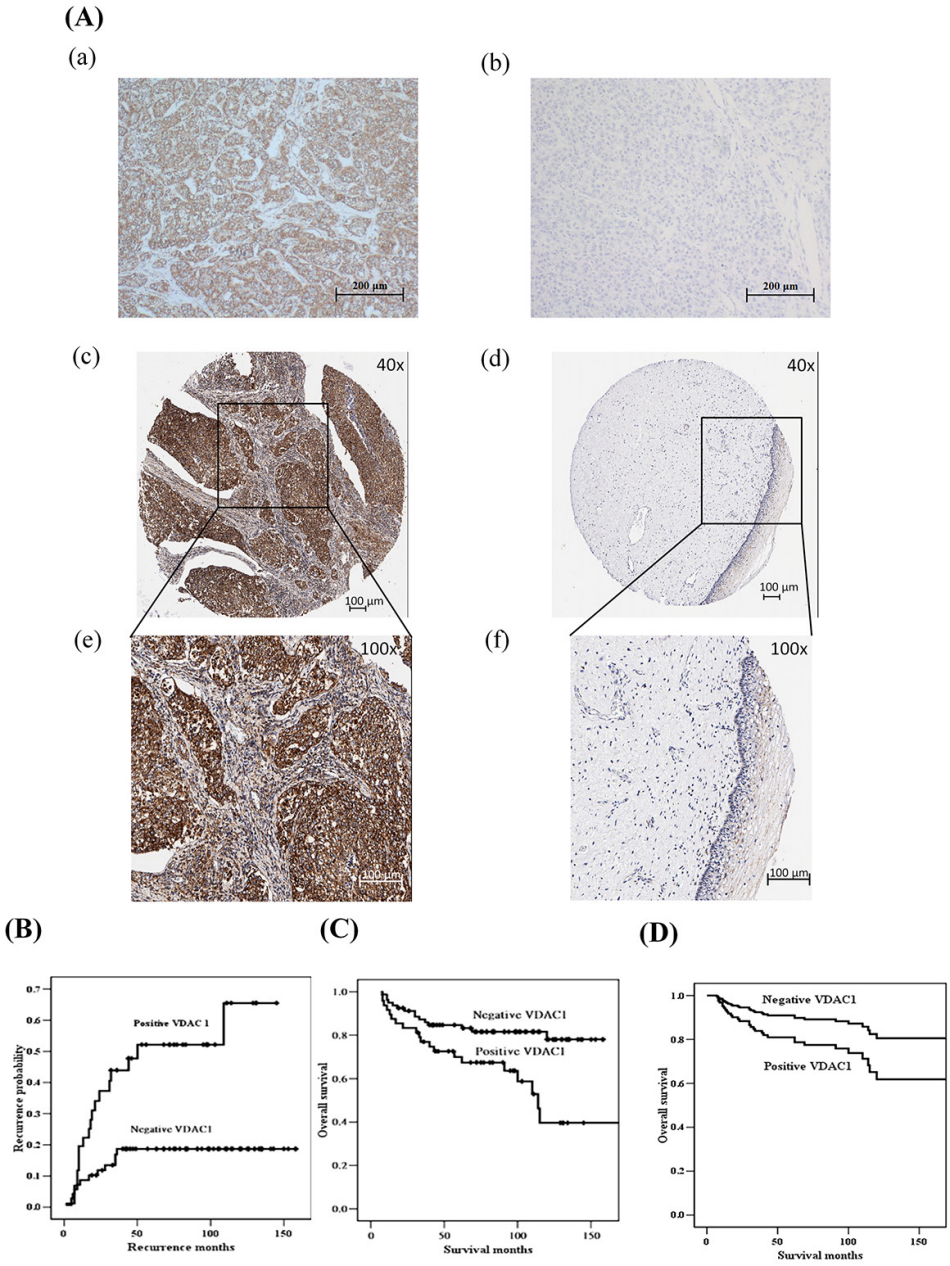
Kaplan-Meier curves for the probability of recurrence and overall survival in cervical cancer patients (n = 150) as well as the survival curves from a Cox proportional hazards model based on the VDAC1 protein expression in cancer tissue cores of the uterine cervix **A.** VDAC1 immunostaining for positive and negative controls as well as representative invasive cancer and normal tissues cores of the uterine cervix. (a) Positive control of VDAC1 staining using liver cells. (b) Negative control of VDAC1 staining using liver cells. (c) and (e) positive immunoreactivity in the cytoplasm of cervical cancer tissues as well as (d) and (f) negative immunoreactivity of cervical normal tissues are photographed at 40× and 100× magnification, respectively. **B.** Cervical cancer patients with positive VDAC1 immunoreactivity had a higher probability of recurrence (*p*=0.0025) compared to those with negative VDAC1 immunoreactivity. **C.** Cervical cancer patients with positive VDAC1 had worse overall survival (*p*=0.0036). The log-rank test was used for statistical comparisons for the Kaplan-Meier curves. **D.** After adjusting for other variables using the Cox proportional hazards model, cervical cancer patients with positive VDAC1 immunoreactivity had a lower overall survival rate than those with negative VDAC1 immunoreactivity (*p*=0.048). VDAC1, voltage-dependent anion channel 1.

We previously found that nm23-H1 was involved in cell proliferation of uterine cervical cancer [1]. Therefore, we investigated the clinical implication of VDAC1 in cervical cancer and found that the immunoreactivity was significantly stronger in cervical cancer tissues than that in normal tissues (*p*<0.001, median: 1.5 vs. 0.5, n=150 vs. 29; Figure 2A). We then investigated the relationship between VDAC1 immunoreactivity of cancer tissues and clinicopathological characteristics of cervical cancer patients. The results showed that cancer tissues with positive VDAC1 immunoreactivity exhibited deep stromal invasion (>10 mm in depth of stromal invasion, *p*<0.001, OR: 4.14 and 95% CI: 1.80-9.88) and large tumor size (>4 cm in diameter, *p*=0.001, OR: 3.77 and 95% CI: 1.60-9.00, Table 2). We also evaluated the correlations of VDAC1 expression with the prognosis of patients with cervical cancer, and found that cervical cancer patients with positive VDAC1 immunoreactivity had a higher probability of recurrence (*p*=0.0025) and lower overall survival (*p*=0.0036) as compared to those with negative VDAC1 (Figure 2B & C). The positive VDAC1 immunoreactivity could predict worse overall survival even after adjusting for other clinicopathological variables (Table 3 and Figure 2D).

**Table 2:** The correlation of voltage-dependent anion channel 1 (VDAC1) immunoreactivity in 150 cancer tissue microarrays with clinicopathological parameters of cancer of the uterine cervix

Clinicopathological variables^a^	VDAC1^b^ (+) (−)	*p* values	OR and 95% CI
Stage		0.334	
I	34 61		1.00
others	22 28		1.41 (0.66–3.00)
Pathologic type		0.080	
squamous cell carcinoma	53 71		1.00
adenocarcinoma	6 19		0.42 (0.13–1.21)
Depth of stromal invasion		<0.001	
≤10 mm	12 46		1.00
>10 mm	40 37		4.14 (1.80–9.88)
Tumor diameter		0.001	
≤4 cm	30 66		1.00
>4 cm	24 14		3.77 (1.60–9.00)
Tumor grade		0.011	
well	3 18		1.00
moderate or poor	47 60		4.70 (1.25–26.11)
Parametrial invasion		0.141	
no invasion	41 73		1.00
invasion	17 17		1.78 (0.76–4.15)
Vaginal invasion		0.150	
no invasion	45 77		1.00
invasion	14 13		1.84 (0.73–4.66)
Pelvic lymph node metastasis		0.526	
negative	43 70		1.00
positive	15 19		1.29 (0.54–2.99)

Statistical analysis: chi-square test.

a Some clinicopathological data could not be collected from the patients with cervical cancer due to incomplete records of medical charts.

b (+): positive immunoreactivity; (−): negative immunoreactivity. The median value of all H scores in 150 cervical cancer cores was determined as the cutoff point to separate VDAC1 positive from VDAC1 negative tissue cores. Semiquantitative H score of VDAC1immunoreactivity was calculated by multiplying the proportion score of stained cells by their immunoreactivity intensity.

OR: odds ratio; CI: confidence interval.

**Table 3:** Univariate and multivariate analyses of the influence of clinical parameters and voltage-dependent anion channel 1 (VDAC1) on survival of patients with cancer of the uterine cervix

Clinicopathological and VDAC1 variables^a^	Case number	5-year survival rate (%)	Hazard ratio	95% confidence interval	*p* value
**Univariate analysis**					
Stage					
I	82	83.6	1.00	Reference	0.25
others	45	70.7	1.47	0.75-2.88	
Pathologic type					
squamous cellcarcinoma	107	82.7	1.00	Reference	0.18
adenocarcinoma	21	60.7	1.72	0.78-3.79	
Depth of stromal invasion					
≤10 mm	49	98.0	1.00	Reference	<0.001
>10 mm	70	67.0	8.62	2.63-28.6	
Tumor diameter					
<4 cm	81	90.0	1.00	Reference	0.0068
>4 cm	36	60.9	2.56	1.26-5.21	
Tumor grade					
well	16	93.8	1.00	Reference	0.090
moderate or poor	94	78.4	4.78	0.65-35.71	
Parametrial invasion					
no invasion	95	85.9	1.00	Reference	0.003
invasion	32	59.4	2.65	1.36-5.18	
Vaginal invasion					
no invasion	103	80.2	1.00	Reference	0.44
invasion	25	75.8	1.35	0.63-2.88	
Pelvic lymph node metastasis					
negative	96	84.0	1.00	Reference	0.011
positive	30	62.2	2.38	1.19-4.76	
VDAC1 immunoreactivity					
negative	80	84.7	1.00	Reference	0.0036
positive	48	70.0	2.61	1.33-5.12	
**Multivariate analysis**					
VDAC1 immunoreactivity					
negative	80	84.7	1.00	Reference	0.048
positive	48	70.0	2.22	1.01-4.90	
Depth of stromal invasion					
≤10 mm	49	98.0	1.00	Reference	0.002
>10 mm	70	67.0	6.79	2.04-22.6	

Statistical analysis: Kaplan-Meier product limit method and univariate and multivariate Cox regression models.

a Some clinicopathological data could not be collected from the patients with cervical cancer due to incomplete records of medical charts.

### Reduction in VDAC1 expression and change in cell growth in the SiHa and CaSki cervical cancer cells with VDAC1 gene silencing

**Figure 3:**
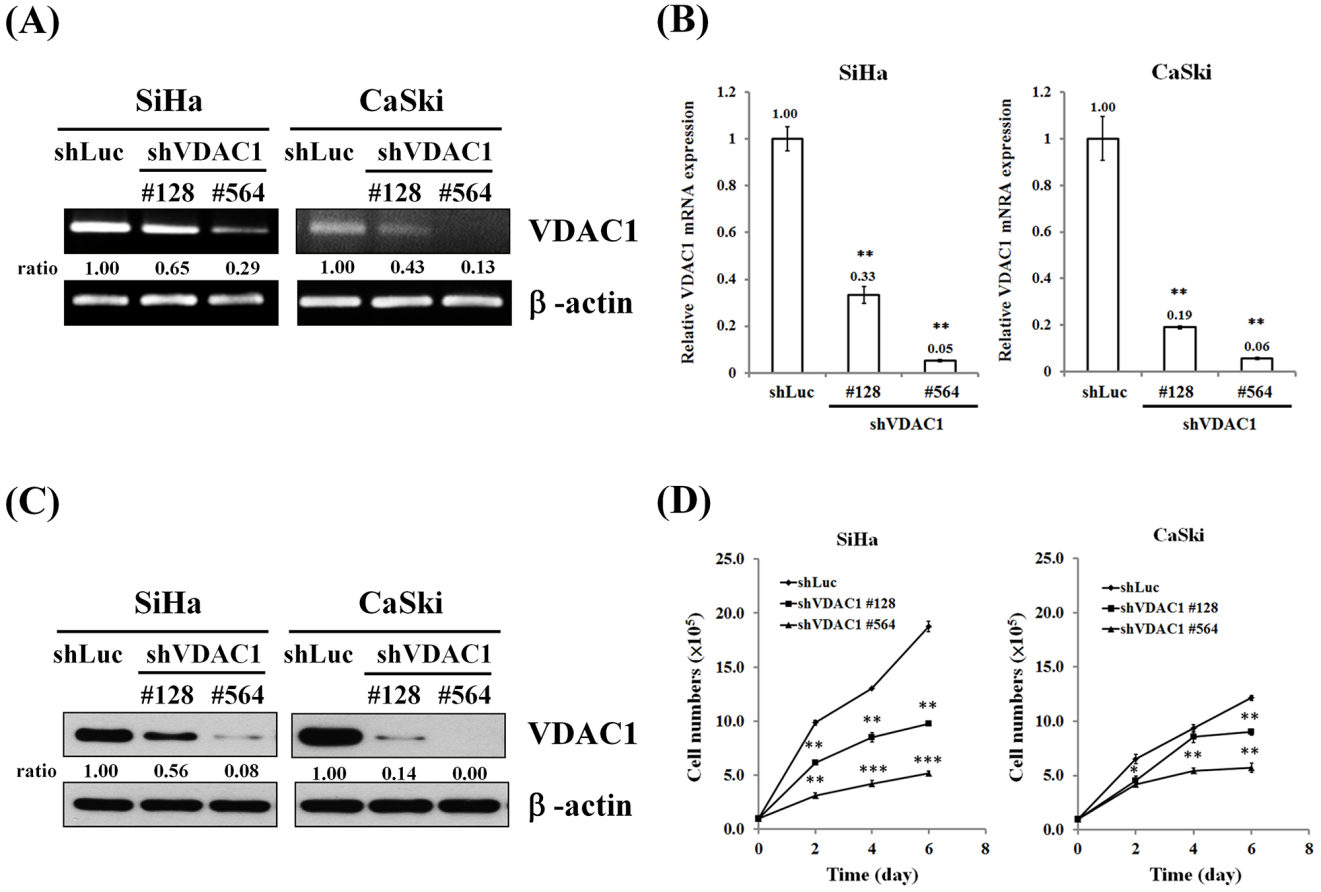
Reduced cell growth after the VDAC1 gene had been silenced in SiHa and CaSki cervical cancer cells SiHa and CaSki cells (5×10^5^ cells/6-cm dish) were infected with lentiviruses carrying shVDAC1 #128, #564 or vector control (shLuc). The RNA levels were determined by **A.** RT-PCR and **B.** real-time PCR. β-actin was used as the internal control. The relative ratios of VDAC1/β-actin are shown. **C.** Western blotting was used to detect the VDAC1 expression in SiHa or CaSki shVDAC1 #128, #564 and shLuc cells. β-actin was used as the internal control. The relative ratios of VDAC1/β-actin are shown. **D.** SiHa or CaSki shVDAC1 #128, #564 or shLuc cells (1 × 10^5^ cells/6-cm dish) were seeded and then analyzed for growth curves for 2, 4 and 6 days by counting cell numbers. All values are means ± SD from at least three independent experiments. *p<0.05, **p<0.01, ***p<0.001.

We next investigated the influence of VDAC1 gene silencing on the growth and metastatic potential of cervical cancer cells. SiHa and CaSki cervical cancer cells in which the VDAC1 gene had been silenced were established, and reduced mRNA levels and protein contents of VDAC1 were confirmed in these cancer cells (Figure 3A, B and C). We found that the cell growth was reduced after VDAC1 gene silencing in SiHa and CaSki cervical cancer cells (Figure 3D and supplemental Figure 1A, B and C).

### Changes in MMP, cell cycle, autophagy and ROS after VDAC1 gene silencing in SiHa and CaSki cervical cancer cells

**Figure 4:**
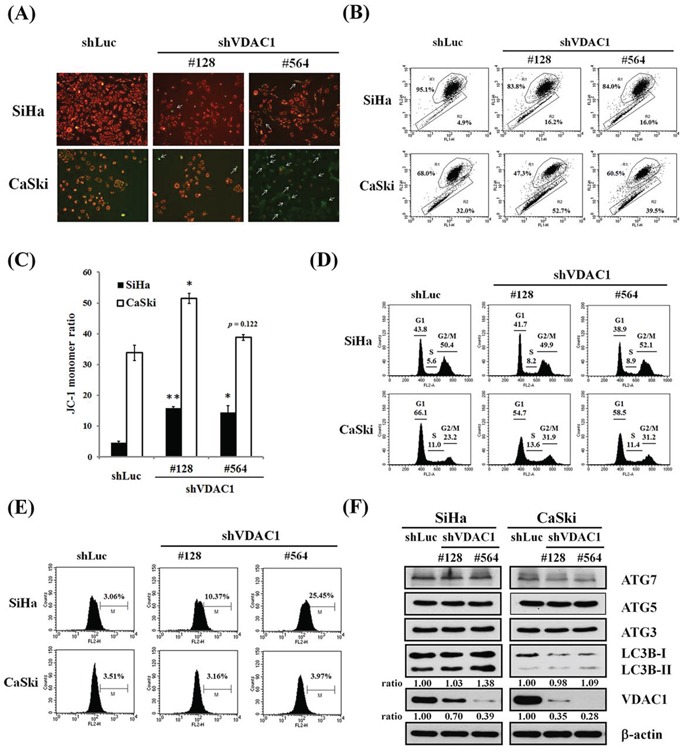
The effects of shVDAC1 on MMP, cell cycle, ROS production and autophagy **A.** Representative images of SiHa and CaSki shVDAC1 #128, #564 and shLuc cells stained with JC-1 (magnification, ×100). White arrows indicate green fluorescence (monomeric JC-1). **B.** MMP measurements by flow cytometry and the intensity of red fluorescence (R1) and green fluorescence (R2) of JC-1. **C.** Bar graph shows changes in quantification in JC-1 fluorescence as detected by flow cytometry assay. The JC-1 monomer ratio was increased in VDAC1 gene-silenced SiHa or CaSki cervical cancer cells compared to their control counterparts. Data are presented as mean ± SD. *p<0.05, **p<0.01. **D.** The cell cycle of SiHa and CaSki shVDAC1 #128, #564 or shLuc cells was analyzed by propidium iodide staining using flow cytometry. No significant changes were found in the VDAC1 gene-silenced cancer cells. **E.** Increased ROS generation was found in the VDAC1 gene-silenced cervical cancer cells, and especially in SiHa shVDAC1 #128 and #564 cancer cells. The cells were stained with 10 μM H2DCFDA for 30 minutes. ROS production was measured by flow cytometry. **F.** The protein levels were determined by Western blotting. β-actin was used as the internal control. The relative ratios of LC3B-II/β-actin and VDAC1/β-actin are shown. The VDAC1 gene was silenced using lentiviruses carrying shVDAC1 #128 and #564 in SiHa and CaSki cervical cancer cells. Control vector: shLuc. VDAC1, voltage-dependent anion channel 1; MMP, mitochondrial membrane potential; ROS, reactive oxygen species; Atg, autophagy-correlated protein; LC3, microtubule-associated protein light chain 3.

In the SiHa and CaSki cervical cancer cells in which the VDAC1 gene had been silenced, the presence of JC-1 monomer in the cytoplasm, as detected by green fluorescence (white arrow in Figure 4A), meant a low MMP in the cervical cancer cells, and the presence of J-aggregates, as exhibited by red fluorescence in the mitochondria, indicated living cancer cells. In these cancer cells, MMPs were reduced, as demonstrated by an increase in JC-1 monomer ratio (Figure 4B). The JC-1 monomer ratio was increased in VDAC1 gene-silenced SiHa or CaSki cervical cancer cells compared to their control counterparts (Figure 4C). Nevertheless, there seemed to be no significant progression in the cell cycle in these VDAC1 gene-silenced cancer cells (Figure 4D). However, ROS generation was significantly increased, and especially in the SiHa shVDAC1 #128 and #564 cancer cells (Figure 4E). Moreover, there was no significant change in autophagy-associated proteins in these VDAC1 gene-silenced cancer cells (Figure 4F).

### Reduced cell motility and migration in VDAC1 gene-silenced cervical cancer cells

**Figure 5:**
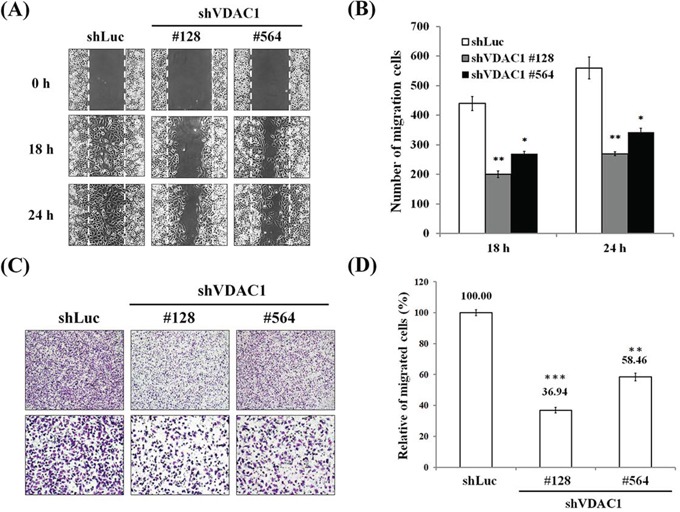
Reduced cell migration in cervical cancer cells in which the VDAC1 gene had been silenced **A.** Wound healing arrays were performed on SiHa shVDAC1 #128, #564 and shLuc cells (2 × 10^4^ cells/well of culture inserts). Images were captured at the indicated times after the initial wound in the top panels (magnification, ×100). **B.** Cell motility was significantly reduced after the VDAC1 gene had been silenced in SiHa cervical cancer cells. The number of cells that had migrated were counted under a microscope. **C.** Reduced cell migration using a Boyden chamber with a polycarbonate membrane (12-μm pore size) after the VDAC1 gene had been silenced in SiHa cervical cancer cells. A representative microscopic image for each condition is shown (magnification, ×40 and ×100). **D.** Cell migration was significantly reduced after the VDAC1 gene had been silenced in SiHa cervical cancer cells. The migration rate was determined by counting the cells that had migrated through the polycarbonate membrane and was expressed as the relative percentage to those with the control vector (shLuc, set to 100%). All data are representative of at least three different experiments. Values are expressed as mean±SD. The VDAC1 gene was silenced using lentiviruses carrying shVDAC1 #128 and #564 in SiHa cervical cancer cells. VDAC1, voltage-dependent anion channel 1. *p<0.05, **p<0.01, ***p<0.001.

The cell motility was found to be significantly reduced with a wound healing assay (Figure 5A) after the VDAC1 gene had been silenced in SiHa cervical cancer cells with lentiviruses carrying shVDAC1 #128 and #564 (Figure 5B). Similarly, cell migration was also significantly reduced in a Boyden chamber assay in the SiHa shVDAC1 #128 and #564 cancer cells (Figure 5C and 5D).

**Figure 6:**
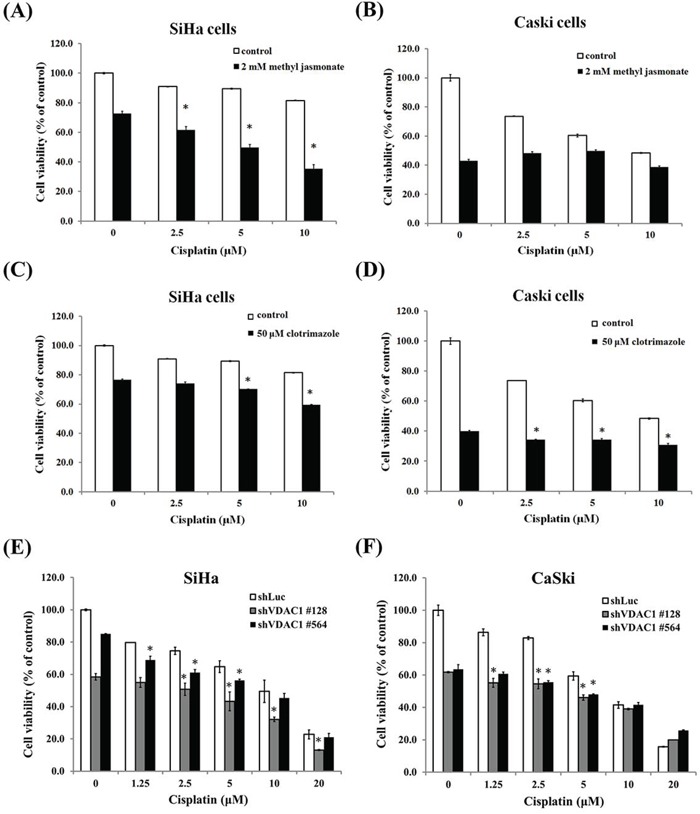
The effects of shVDAC1 and compounds disrupting VDAC1 and hexokinase interactions on the cytotoxicity of cisplatin in SiHa and CaSki cervical cancer cells **A.** Two mM of methyl jasmonate significantly enhanced the cell toxicity of cisplatin at concentrations of 2.5, 5 and 10μM in the SiHa cervical cancer cells. **B.** Two mM methyl jasmonate enhanced the cell toxicity of cisplatin at concentrations of 0, 2.5, 5 and 10μM in the CaSki cervical cancer cells but without statistical significance. **C.** FiftyμM of clotrimazole significantly enhanced the cell toxicity of cisplatin at concentrations of 5 and 10μM in the SiHa cervical cancer cells. **D.** FiftyμM of clotrimazole significantly enhanced the cell toxicity of cisplatin at concentrations of 2.5, 5 and 10μM in the CaSki cervical cancer cells. **E.** Cell cytotoxicity was enhanced when the VDAC1 gene was silenced at concentrations of 1.25-20μM of cisplatin in the SiHa shVDAC1 #128 and #564 cancer cells. **F.** Cell cytotoxicity was enhanced when the VDAC1 gene was silenced at concentrations of 1.25-5μM of cisplatin in the CaSki shVDAC1 #128 and #564 cancer cells. The VDAC1 gene was silenced using lentiviruses carrying shVDAC1 #128 and #564 in SiHa and CaSki cervical cancer cells. VDAC1, voltage-dependent anion channel 1. *p<0.05.

Furthermore, we added reagents that could dissociate the binding of hexokinase and VDAC1, including methyl jasmonate and clotrimazole, to cisplatin in the SiHa and CaSki cervical cancer cells to detect their effects on cell viability. We found that these reagents significantly enhanced cell cytotoxicity to these cancer cells at different concentrations of cisplatin (Figure 6A–D). We then checked the shVDAC1 effects on hexokinase expression in CaSki and SiHa cervical cancer cells. When VDAC1 was silenced in SiHa and CaSki cells, the total cell lysates (50 μg of total proteins) protein levels of hoxokinase 2 exerted no difference in SiHa and CaSki shVDAC1 #128, #564 cells as compared to their parental cancer cells (data not shown). This implies that the amount of hexokinases those bind to VDAC1 but not cell total hexokinases is decreased (the direct evidence may be demonstrated in the future study). We also silenced the VDAC1 gene in SiHa and CaSki cancer cells to examine the influence of VDAC1 on cisplatin cytotoxicity, and found that when the VDAC1 gene had been silenced, the cell cytotoxicity of cisplatin was significantly enhanced at different concentrations of cisplatin (Figure 6E and 6F). In addition, the cell viability was reduced more than 70% at a concentration of 5 μM cisplatin in CaSki shVDAC1 #481 and #509 cervical cancer cells and SiHa shVDAC1 #128 and #564 cancer cells were resistant to cisplatin, we therefore showed the reduction of MMP with cisplatin at a concentration of 20 μM in the VDAC1 gene-silenced SiHa #481 and #509 and with cisplatin at a concentration of 5 μM in CaSki #128 and #564 cervical cancer cells compared to their control counterparts (supplemental Figure 2). The JC-1 monomer ratio was also increased in the VDAC1 gene-silenced SiHa #481 and #509 and CaSki #128 and #564 cervical cancer cells with cisplatin.

## DISCUSSION

Cell growth has also been reported to be increased in nm23-H1-silenced SiHa cancer cells [1]. After the nm23-H1 gene was silenced, the expression of VDAC1 was elevated in the mRNA and protein levels in SiHa and CaSki cervical cancer cells in this study. It has been reported that silencing of nm23 in hepatoma and colon carcinoma cell lines resulted in upregulation of the membrane associated matrix metalloproteinase, increased Rac1 signaling and activation of several pro-invasive signaling pathways including mitogen-activated protein kinase/stress-activated protein kinases [25]. Cai et al. found that nucleoside diphosphate kinase (NDPK, nm23) activates transient receptor potential-vanilloid-5 (TRPV5) channel activity and Ca^2+^ flux [26]. It is physiologically relevant to Ca^2+^ reabsorption in vivo, as short hairpin RNA knockdown of NDPK leads to decreased TRPV5 channel activity. Increased Ca^2+^ levels up-regulate VDAC1 expression [27]. Silencing of VDAC1 decreases mitochondrial membrane potential and increases ROS production. Reducing VDAC1 inhibits cancer cell growth and tumor development in vivo [28]. We therefore hypothesized that shnm23-H1 increased the VDAC1-dependent cell growth and migration through up-regulating Ca^2+^ in cervical cancer cells. Hanahan et al. proposed that deregulating cellular energetic is an important hallmark during the multistep development of human cancer [29]. Functions of VDAC1 in metabolism and energy homeostasis are reflected by its facilitation of the transport of ions, nucleotides and other metabolites across the outer mitochondrial membrane [30–32]. Compared to normal counterparts, our findings showed that the VDAC1 immunoreactivity was significantly stronger in cervical cancer tissues. This finding is supported by the results of Shoshan-Barmatz et al., in which over-expression of VDAC1 in cancer tissues of the cervix, lung, thyroid and ovary was clearly demonstrated using cancer tissue arrays and immunohistochemistry relative to non-cancerous tissues [33].

This study revealed that patients with cervical cancer whose cancer tissues exhibited positive VDAC1 immunoreactivity had a higher risk of recurrence and poor overall survival. Even after adjusting for other clinicopathological characteristics in multivariate analysis, these patients still had a poorer overall survival. Ko et al. also demonstrated that the VDAC1-associated gene signature is a robust predictive biomarker of recurrence-free survival in breast, colon, and lung cancers, and that it is independent of standard clinical and pathological prognostic factors [34]. Grills et al. reported that VDAC1 over-expression predicted a shorter time to recurrence and overall survival in non-small cell lung cancer [35].

After silencing VDAC1 in SiHa and CaSki cervical cancer cells, the cell growth was significantly inhibited. Abu-Hamad et al. reported that silencing of the VDAC1 gene in T-Rex-293 led to reduced ATP production and a decrease in cell growth, and that cells in which the VDAC1 expression was reduced by approximately 90% proliferated extremely slowly [36]. VDAC1 is expressed in all mammalian cells and at high levels in cancer cells [37]. Koren et al. demonstrated that HeLa cervical cancer cells with shRNA-VDAC1 proliferated much slower than control cells, indicating that VDAC1 expression is essential for normal growth of HeLa cancer cells [38]. We further found that the inhibition of cell growth may be through a reduction in MMP.

The VDAC channel has voltage-dependent conductance and displays ion selectivity. At low voltages (10 mV), the channel is stable in a long-lived open state [39, 40]. In many physiopathological models, prolonged opening of the permeability transition pore, which is composed of VDAC, adenine nucleotide translocase and cyclophilin D, has been reported to lead to mitochondrial permeability transition, mitochondrial matrix swelling and local rupture of the outer mitochondrial membrane thereby resulting in cell death [15, 16]. These events are accompanied by the dissipation of mitochondrial transmembrane potential.

ROS generation was significantly increased in SiHa shVDAC1 #128 and #564 but not apparently in CaSki shVDAC1 #128 and #564 cells. To confirm the difference of cervical cancer cell types for ROS generation, we detected the effects of shVDAC1 on ROS metabolizing enzyme in SiHa and CaSki shVDAC1 #128 and #564 cell lines. Among these enzymes, we found that the expression levels of catalase were elevated after the VDAC1 gene was silenced in CaSki but none of the ROS metabolizing enzymes expression was increased in SiHa shVDAC1 #128 and #564 cells (supplemental Figure 3). The amount of catalase was increased to scavenge the hydrogen peroxide by catalyzing the hydrogen peroxide to water and oxygen in Caski shVDAC1 #128 and #564 cells. ROS has been suggested to activate cytochrome c release from mitochondria [41], and over-expression of hexokinase 1 or hexokinase 2 has been reported to inhibit ROS release from the mitochondria to the cytosol [42]. The Warburg effect has been noted in cancer cells, characterized by a high rate of glycolysis resulting in enhanced lactate generation [43, 44]. Hexokinase catalyzes the phosphorylation of glucose to glucose-6-phosphate, which is the first step of glycolysis. In many cancers including lymphoma, colon, prostate and breast cancers, the remarkable propensity of malignant cells for high glycolysis activity frequently relies on the over-expression of hexokinase 1 and/or hexokinase 2 [43–47]. Hexokinase isozymes are capable of binding to the outer mitochondrial membrane, and specifically to VDAC [10]. By binding to VDAC1, hexokinase acquires increased levels of mitochondria-bound hexokinase in cancer cells, which interferes with VDAC1 Bax or Bak interactions and prevents cytochrome c release, and may acquire direct access to the mitochondrial ATP pool for phosphorylation of glucose, subsequently protecting against mitochondria-induced cell death [45, 48]. Grill et al. also demonstrated that the main combined activity of the VDAC1 gene was focused on ATP and nucleotide binding [35]. A reduction in the interactions between hexokinase and VDAC1 can lead to increased H_2_O_2_ generation, thereby activating cell death [42, 49]. Therefore, in cervical cancer cells in which the VDAC1 gene had been silenced, it prevented the interaction of hexokinase and VDAC1, subsequently reduced glycolysis and energy production and then increased the generation of ROS and inhibited cell growth. Moreover, autophagy and cell cycle progression were not changed in VDAC1 silencing cells.

Tumor cells produce lactic acid via glycolysis which is transported out of the cell creating low pH conditions. An acidic microenvironment protects tumors against attack by the immune system while causing damage to normal surrounding cells, thus preparing them for invasion [29]. When the VDAC1 gene is silenced, hexokinase cannot bind to VDAC1 and cannot acquire direct access to the mitochondrial ATP pool for phosphorylation of glucose and energy production, thereby influencing the Warburg effect. This may explain why the ability of cell migration was significantly reduced in the SiHa shVDAC1 #128 and #564 cells.

Cancer therapy strategies aimed at VDAC include siRNA altering the normal functioning of cancer cells leading to growth arrest [33]. We found that the cytotoxicity of cisplatin was significantly enhanced in the SiHa and CaSki cervical cancer cells in which the VDAC1 gene had been silenced. However, Tajeddine et al. reported that a decrease in cisplatin-induced death was correlated with a decrease in mitochondrial trans-membrane potential dissipation in siRNA-VDAC1-expressing cells [50]. An increased expression of VDAC1 has been reported to be a promising strategy to improve DNA cross-linker-induced chemotherapy [51]. In addition to VDAC1 gene knockdown, we also found that the reagents methyl jasmonate and clotrimazole, which could disrupt the interactions of VDAC1 and hexokinase, also significantly enhanced the cytotoxicity of cisplatin. They have been reported to exert this effect through binding to hexokinase and detaching it from its binding site with mitochondria in many cancer cell types [23, 52]. Based on our findings, therapeutic strategies should be offered using VDAC1 as a target to reduce cell growth and migration, enhance the synergistic therapeutic efficacy of cisplatin and reduce dose-limiting toxicity.

## MATERIALS AND METHODS

### Cell culture

SiHa and CaSki cancer cell lines of the uterine cervix and human embryonic kidney cell line 293T were obtained from the American Type Tissue Culture Collection (ATCC; Rockville, MD, USA). SiHa and 293T cells were cultured in Dulbecco's Modified Eagle Medium (DMEM, Invitrogen, Grand Island, NY) containing 10% heat-inactivated fetal bovine serum (FBS, Gibco, Grand Island, NY). CaSki cells was cultured in Roswell Park Memorial Institute (RPMI) 1640 (Gibco, Grand Island, NY) containing 10% heat-inactivated FBS.

### shRNA and lentiviral production

The lentiviral expression system and pLKO-AS1-puromycin (puro) plasmid encoding shRNAs were obtained from the National RNAi Core Facility at the Institute of Molecular Biology/Genomic Research Center, Academia Sinica, Taiwan. Individual clones were identified by their unique TRC number, including shVDAC1 #128 (TRCN0000029128, responding sequence, 5′-CAAGTACAGATGGACTGAGTA-3′), shVDAC1 #481 (TRCN0000297481, responding sequence, 5′-GCAGTTGGCTACAAGACTGAT-3′), shVDAC1 #509 (TRCN0000278509, responding sequence, 5′-GCTATGGATTTGGCTTAATAA-3′), shVDAC1 #564 (TRCN0000278564, responding sequence, 5′-GCTTGGTCTAGGACTGGAATT-3′), shnm23-H1 #61 (TRCN0000010061, responding sequence, 5′-TCCGCCTTGTTGGTCTGAAAT-3′), shnm23-H1#62 (TRCN0000010062, responding sequence, 5′-CCGCCTTGTTGGTCTGAAATT-3′) and shLuc (TRCN0000072246, responding sequence, CAAATC ACAGAATCGTCGTAT).

### Two-dimensional (2D) gel electrophoresis and mass spectrophotometry (MS) analysis

2D gel electrophoresis was performed following the modified protocol of GE Healthcare Life Sciences (Sweden). The gels were stained overnight in SYPRO Ruby protein gel stains (Life Technologies, Inc., Carlsbad, California, USA) and scanned with an Image Scanner (AlphaImager 2000, Alpha Innotech, San Leandro, CA, USA). MS analysis was performed by the Instrument Center of Chung Shan Medical University, Taiwan.

### Isolation of RNA, reverse transcription-PCR and quantitative real time PCR

Total cellular RNA was extracted from cells using RareRNA (Genepure Technology, Taiwan) according to the manufacturer's protocol. The following primers were used for PCR: VDAC1, forward 5′-CCACCCACGTATGCCGATCTTG-3′ and reverse 5′-GTCAGGCCGTACTCAGTCCATC-3′; nm23-H1: forward, 5′-TGCTGCGAACCACGTGGGTCCCGG-3′ and reverse, 5′-TCATTCATAGATCCAGTTCTGAGCA-3′; β-actin: forward, 5′-CAGGGAGTGATGGTGGGCA-3′ and reverse, 5′-CAAACATCATCTGGTCATCTTCTC-3′. Real time PCR was performed using an ABI StepOnePlus real time PCR system with gene-specific primers and Smart Quant GreenMaster Mix (Protech Technology Enterprise Co., Taipei, Taiwan).

### Western blot analysis

Anti-VDAC1 (Abgent, AP6627a, San Diego, CA, USA), anti-nm23-H1 (Novocastra, Leica Biosystems, UK), anti-β-actin (Sigma, St. Louis, MO, A5441) and autophagy antibody sampler kit (Cell Signaling Technologies, 4445, Danvers, MA, USA) antibodies were used to detect VDAC1, nm23-H1, β-actin, ATG7, ATG5, ATG3 and LC3B. The complete protocol for Western blot analysis has been described previously [53].

### Association of immunohistochemical expression of VDAC1 with the prognosis of cervical cancer patients using tissue microarrays

Three formalin-fixed, paraffin-embedded tissue microarrays (MaxArray tissue cores, Zymed, South San Francisco, California), consisting of invasive cancer and normal tissue specimens of the uterine cervix were constructed. These tissue specimens were collected from histopathological paraffin blocks from the Pathology Department of Chung Shan Medical University. A total of 150 patients with cervical cancer were recruited consecutively between March 1999 and May 2010. They were staged according to the International Federation of Gynecology and Obstetrics (FIGO) Classification [54] and received standard treatment protocols at the Department of Obstetrics and Gynecology, Chung Shan Medical University Hospital, Taiwan. Tissue microarray sections were incubated with anti-VDAC1 antibodies [1:600 dilution, Anti-VDAC1/Porin antibody-Mitochondrial Loading Control (ab15895), Abcam Inc., Cambridge, MA]. A semi-quantitative H score of VDAC1 immunoreactivity was determined by multiplying the proportional score of stained cells by their immunoreactivity intensity [55]. Investigation has been conducted in accordance with the ethical standards and according to the Declaration of Helsinki and according to national and international guidelines. This study was approved by the Chung Shan Medical University Hospital Institutional Review Board (CSMUH IRB CS12219) and informed consent was obtained from each patient.

### Cell growth and cell viability assay

Around 1 × 10^5^ of SiHa or CaSki shVDAC1 #128, #481, #509, #564 and shLuc cells were seeded onto 6-cm dishes. The number of cells was counted on days 3, 5, and 7 to plot growth curves. Cell viability experiments were carried out using 3- (4,5-cimethylthiazol-2-yl)-2,5-diphenyl tetrazolium bromide (MTT, Sigma, M5655, Sigma Chemical Co., St. Louis, MO) assays. SiHa or CaSki cervical cancer cells, including VDAC1 gene silencing and control vector transfection in these cancer cells, were seeded onto a 96-well microculture plate at 5000 cells/well and allowed to attach overnight. The next day, the cells were exposed to different concentrations of cisplatin (0, 1.2, 2.5, 5, 10 or 20 μM) with or without methyl jasmonate (2 mM) or clotrimazole (50 μM) in DMEM medium supplemented with 10% FBS and incubated for 48 hours. The medium was replaced with fresh medium containing MTT (0.2 mg/ml) and the plates were incubated for another 3 hours. The medium was then removed and dimethyl sulfoxide was added to dissolve the MTT formazan crystals. Absorbance of the color was measured at 570 nm, and cell viability was calculated as the percentage of viable cells in the total population. In addition, Cell Counting Kit-8 (CCK-8; Sigma, M5655, Sigma Chemical Co., St. Louis, MO) assay was also done and the absorbance of the color was measured at 450 nm.

### Detection of mitochondrial membrane potential (MMP) by JC-1 dye

JC-1 is a mitochondrial dye that stains mitochondria in living cells in a membrane potential-dependent fashion. JC-1 monomer, which is favored at a low MMP and is present in cytoplasm, is in equilibrium with so called J-aggregates, which are favored at a higher MMP and present in mitochondria. The monomer JC-1 has green fluorescence (lem=527 nm), while J-aggregates have red fluorescence (lem=590 nm). Therefore, it is possible to calculate the fluorescence ratio to study MMP as detected in flow cytometry. One μM JC-1 fluorescent dye (Invitrogen, T3168, Grand Island, NY) was added to the cells for 30 minutes of incubation, and then the red (aggregated JC-1) and green (monomeric JC-1) fluorescence signals were observed by fluorescence microscopy and analyzed by flow cytometry (BD Biosciences, San Jose, CA, USA).

### Detection of intracellular reactive oxygen species (ROS)

The presence of ROS in the cells was monitored using 10 μM of the fluorescent ROS indicator H_2_DCFDA (Invitrogen, D399, Grand Island, NY) for 30 minutes. The cells were harvested and analyzed by flow cytometry.

### Wound healing and migration assays

Seventy μL of SiHa or CaSki cells (3 × 10^5^ cells/ml) were seeded and cultured onto a 24-well plate with culture inserts (ibidi GmbH, Munich, Germany). The cells were photographed under a light microscope at the indicated times. The migration assay was performed using a modified Boyden chamber with an 8-μm pore size polycarbonate membrane. Migrated cells on the lower surface of the membrane were fixed and stained with 20% Giemsa stain (Merck, Darmstadt, Germany).

### Statistical analysis

We associated the expression of VDAC1 with the clinicopathological characteristics of the patients with cervical cancer using the chi-square test. The median value of all H scores of the tumor cells for VDAC1 immunohistochemical expression in cervical cancerous cores was selected as the cutoff point to separate VDAC1 positive tissue cores from negative cores. *P* values, odds ratios (ORs) and 95% confidence intervals (CIs) were calculated using WinPepi Software, version 10.0. Kaplan-Meier curves were plotted for the cervical cancer patients based on the VDAC1 expression for the probability of recurrence or overall survival between primary surgery and recurrence or death or the end of the study (May 31, 2012). Kaplan-Meier product-limit estimate and univariate and multivariate Cox regression models were used to assess the prognostic value of VDAC1 and clinical parameters with or without adjustments for VDAC1 expression and clinicopathological variables, and curves of the probability of recurrence and overall survival were plotted. Comparisons of the mRNA levels from quantitative PCR, cell growth, JC-1 monomer ratio and cell migration and the influence of cell viability from VDAC1 contents and reagents on cervical cancer cells were evaluated using the independent Student's *t* test. All statistical analyses were performed using SPSS statistical software (version 11.0; SPSS, Inc., Chicago, IL). All statistical tests were two-sided, and a *p* value of less than 0.05 was considered to be statistically significant.

## SUPPLEMENTARY FIGURES


