# Supplementary material for: Clinical implication of voltage-dependent anion channel 1 in uterine cervical cancer and its action on cervical cancer cells
Source: Oncotarget. 2015 Dec 21;7(4):4210–25. doi: 10.18632/oncotarget.6704 (PMC4826200; doi:10.18632/oncotarget.6704)
Supplement: Supplementary file 1 [file oncotarget-07-4210-s001.pdf]

SUPPLEMENTARY FIGURES

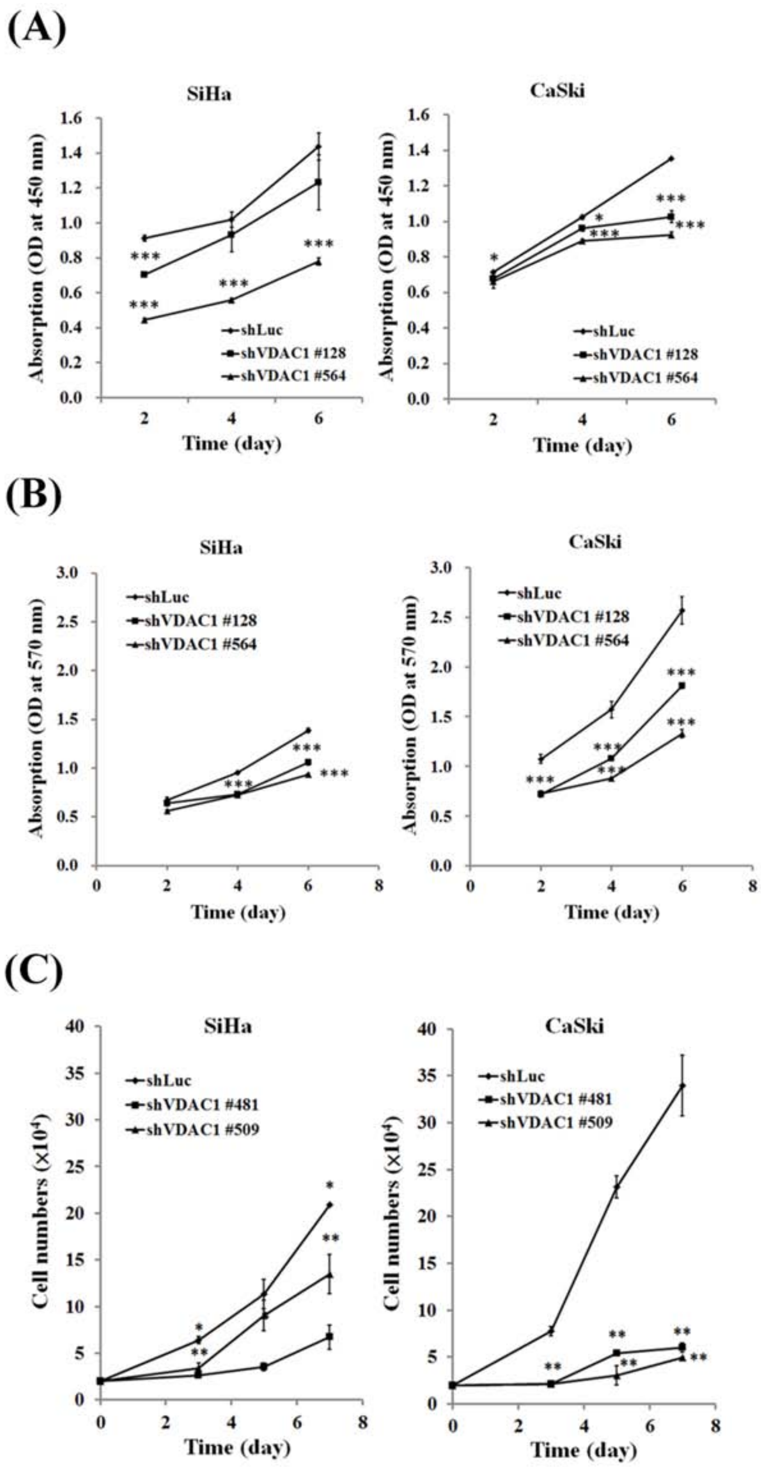

Supplementary Figure S1: Effects of shVDAC1 on cell proliferation and viability in SiHa and CaSki cells. SiHa (left) and CaSki (right) shVDAC1 #128, #564 or shLuc cells (5×10^3/well) were seeded in a 96 well plate and analyzed by **A.** Cell Counting Kit-8 (CCK-8) assay and **B.** MTT [3-(4,5-dimethylthiazol-2-yl)-2,5-diphenyltetrazolium bromide] assay every 48 h under monolayer culture conditions. Each growth curve represents the mean±SD of triplicate experiments. **C.** SiHa and CaSki shVDAC1 #481, #509 or shLuc cells (2×10^4 cells/6cm dish) were seeded and analyzed by counting cell numbers at 3, 5 and 7 days. All values are means ± SD from at least three independent experiments. **p*<0.05, ***p*<0.01, ****p*<0.001.

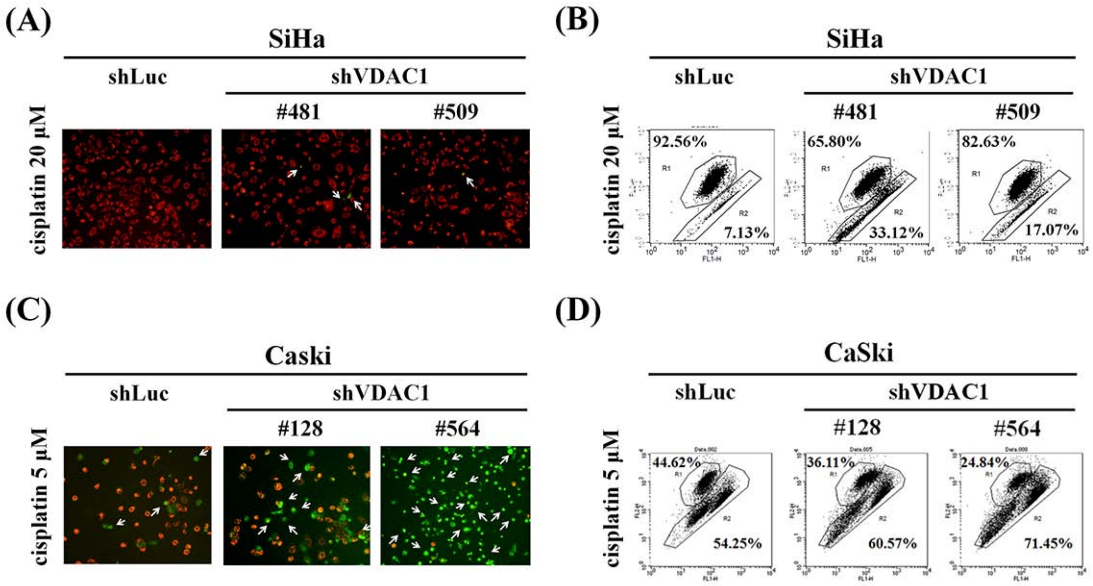

Supplementary Figure S2: Effects of shVDAC1 on MMP in SiHa and CaSki cells. A. SiHa shVDAC1 #481 and #509 and **C.** CaSki shVDAC1 #128, #564 and shLuc cells were treated with cisplatin (5 or 20 μM) for 48 hours. Representative images of SiHa shVDAC1 #481 and #509 and CaSki shVDAC1 #128, #564 and shLuc cells stained with JC-1 (magnification, ×100). White arrows indicate green fluorescence (monomeric JC-1). **B.** SiHa shVDAC1 #481 and #509 and **D.** CaSki shVDAC1 #128, #564 and shLuc cells were treated with cisplatin (5 or 20 μM) for 48 hours. Changes in MMP were assessed by the intensity of red fluorescence (R1) and green fluorescence (R2) of JC-1. VDAC1, voltage-dependent anion channel 1; MMP, mitochondrial membrane potential.

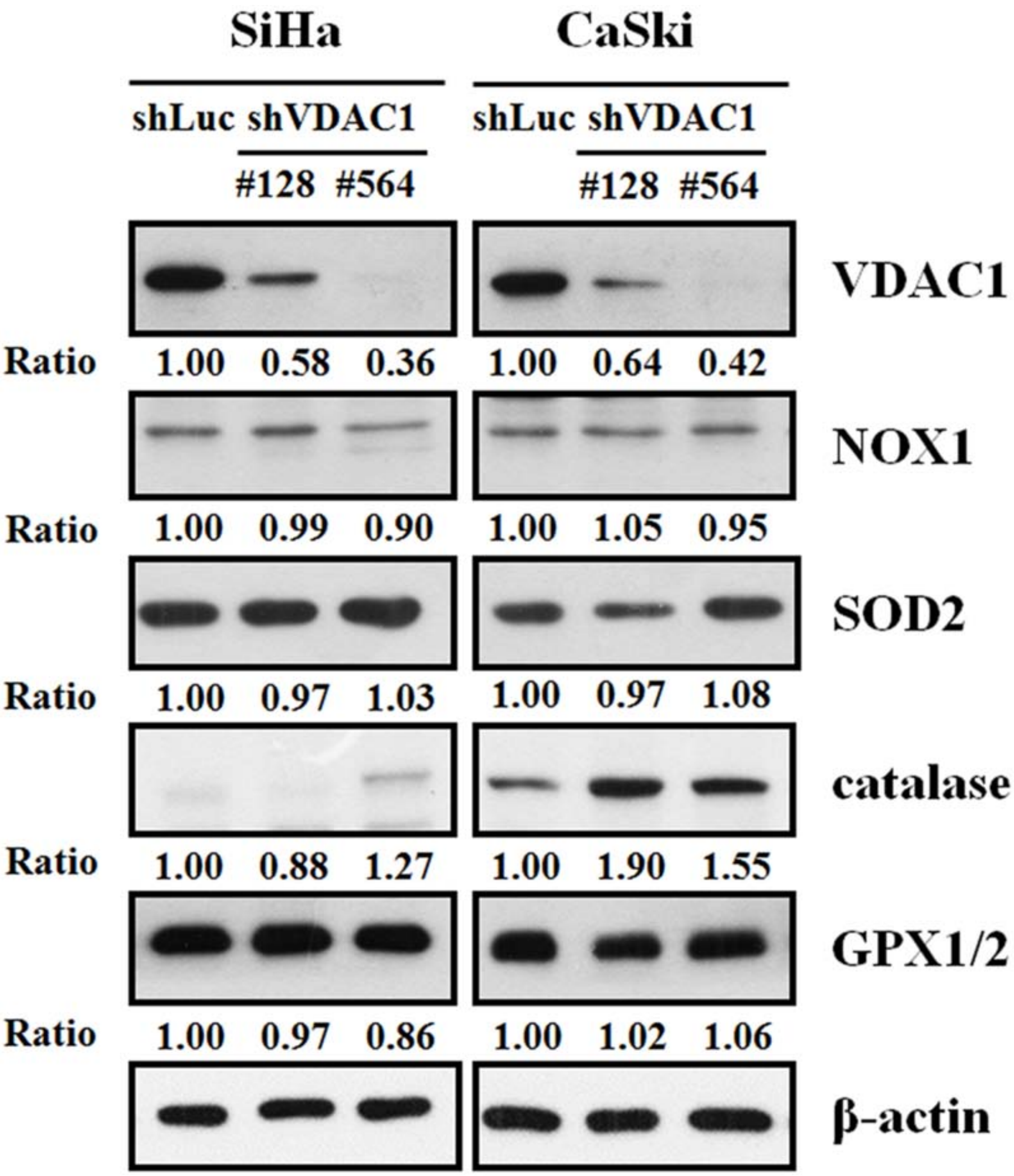

Supplementary Figure S3: Effects of shVDAC1 on the metabolizing enzymes of reactive oxygen species. VDAC1 (voltage-dependent anion channel 1), NOX1 (NADPH oxidase 1), SOD2 (superoxide dismutase 2), catalase and GPX1/2 (glutathione peroxidase 1/2) protein levels were determined on Western blot using protein lysates from parental and VDAC1-knocked down cervical cancer cells. β-actin was used as the internal control. The relative ratios of VDAC1/β-actin, NOX1/β-actin, SOD2/β-actin, catalase/β-actin and GPX1/2/β-actin are shown.
